# Ablation of epidermal RXRα in cooperation with activated CDK4 and oncogenic NRAS generates spontaneous and acute neonatal UVB induced malignant metastatic melanomas

**DOI:** 10.1186/s12885-017-3714-6

**Published:** 2017-11-09

**Authors:** Sharmeen Chagani, Rong Wang, Evan L. Carpenter, Christiane V. Löhr, Gitali Ganguli-Indra, Arup K. Indra

**Affiliations:** 1 Molecular and Cellular Biology Program, OSU, Corvallis, 97331 OR USA; 2 Department of Pharmaceutical Sciences, College of Pharmacy, OSU, Corvallis, 97331 OR USA; 3 Linus Pauling Institute, OSU, Corvallis, OR USA; 4 College of Veterinary Medicine, Oregon State University, Corvallis, Oregon, 97331 USA; 5 Knight Cancer Institute, Oregon Health & Science University (OHSU), Portland, 97239 OR USA; 6 Department of Dermatology, OHSU, Portland, 97239 OR USA

**Keywords:** Keratinocytes, Melanocytes, Acute UVB, Retinoid-X-receptor α (RXRα), Malignant melanoma, Trigenic, Spontaneous melanoma, NRAS^Q61K^, CDK4^R24C/R24C^, Microenvironment

## Abstract

**Background:**

Understanding the underlying molecular mechanisms involved in the formation of cutaneous malignant melanoma is critical for improved diagnosis and treatment. Keratinocytic nuclear receptor Retinoid X Receptor α (RXRα) has a protective role against melanomagenesis and is involved in the regulation of keratinocyte and melanocyte homeostasis subsequent acute ultraviolet (UV) irradiation.

**Methods:**

We generated a trigenic mouse model system (*RXRα* ^*ep−/−*^| *Tyr-NRAS* ^*Q61K*^ | *CDK4* ^*R24C/R24C*^ *)* harboring an epidermal knockout of *Retinoid X Receptor α* (*RXRα* ^*ep−/−*^), combined with oncogenic *NRAS* ^*Q61K*^ (constitutively active RAS) and activated *CDK4* ^*R24C/R24C*^ (constitutively active CDK4). Those mice were subjected to a single neonatal dose of UVB treatment and the role of RXR *α* was evaluated by characterizing the molecular and cellular changes that took place in the untreated and UVB treated trigenic *RXRα* ^*ep−/−*^ mice compared to the control mice with functional RXRα.

**Results:**

Here we report that the trigenic mice develops spontaneous melanoma and exposure to a single neonatal UVB treatment reduces the tumor latency in those mice compared to control mice with functional RXRα. Melanomas from the trigenic *RXRα* ^*ep−/−*^ mice are substantial in size, show increased proliferation, exhibit increased expression of malignant melanoma markers and exhibit enhanced vascularization. Altered expression of several biomarkers including increased expression of activated AKT, p21 and cyclin D1 and reduced expression of pro-apoptotic marker BAX was observed in the tumor adjacent normal (TAN) skin of acute ultraviolet B treated trigenic *RXRα* ^*ep−/−*^ mice. Interestingly, we observed a significant increase in p21 and Cyclin D1 in the TAN skin of un-irradiated trigenic *RXRα* ^*ep−/−*^ mice, suggesting that those changes might be consequences of loss of functional RXRα in the melanoma microenvironment. Loss of RXRα in the epidermal keratinocytes in combination with oncogenic *NRAS* ^*Q61K*^ and *CDK4* ^*R24C/R24C*^ mutations in trigenic mice led to significant melanoma invasion into the draining lymph nodes as compared to controls with functional RXRα.

**Conclusions:**

Our study demonstrates the protective role of keratinocytic RxRα in (1) suppressing spontaneous and acute UVB-induced melanoma, and (2) preventing progression of the melanoma to malignancy in the presence of driver mutations like activated *CDK4* ^*R24C/R24C*^ and oncogenic *NRAS* ^*Q61K*^ *.*

**Electronic supplementary material:**

The online version of this article (10.1186/s12885-017-3714-6) contains supplementary material, which is available to authorized users.

## Background

Malignant melanoma is the deadliest form of skin cancer with approximately 76,000 new cases and an estimated 10,000 deaths in the US this year [1]. Although it arises in both the chronic and non-chronic UV damaged skin, epidemiological studies indicate an increased association with acute rather than chronic sun exposure [2]. Despite the fact that early detection and treatment of the tumor can lead to a high cure rate of patients, malignant melanoma has an unpredictable evolution due its high metastatic ability that is typically non-responsive to current therapies [3]. Melanoma originates in the pigment producing melanocytes, which protect the skin against harmful effects of UV irradiation of the sun. Transformation of melanocytes to cutaneous melanoma is a multistep process called melanomagenesis [4]. This involves formation of a benign nevus and radial proliferation of the melanocytes to the basement membrane in the skin known as radial growth phase followed by the vertical proliferation of the melanocytes called vertical growth phase into the dermis crossing the basement membrane. The tumor is considered invasive at this point and ultimately turns metastatic when the cells enter the bloodstream or lymphatic vessels from which they colonize to other tissues and organs [5]. Keratinocytes are predominantly present in the epidermis and support it through a constant cycle of proliferation and differentiation [6]. Keratinocytes protect the body from UV radiation through transfer of melanin from melanocytes [7], and promote melanocyte proliferation and homeostasis through secretion of paracrine factors [8, 9]. Therefore, understanding the molecular mechanisms behind melanomagenesis is critical for elucidating novel pathways that can be co-opted to aid with diagnosis and treatment strategies.

Retinoid-X-Receptors (RXRs) subtypes α, β, and γ are members of the steroid hormone superfamily of nuclear receptors (NR), and play an important role in gene regulation mediating biological processes such as development, differentiation, and homeostasis [10]. RXRs function as ubiquitous DNA-binding transcription factors and heterodimerize with some 15 NR family members [10, 11], which results in synergistic induction of gene expression via multiple signaling pathways by interacting with several coactivators and corepressors [12]. Previously, we have shown that by deleting RXRα protein specifically in the epidermal keratinocytes via Cre-LoxP technology alters paracrine signals to the melanocytes enhancing melanomagenesis [13, 14]. This deletion also increases melanocyte proliferation and leads to DNA damage repair that is flawed following acute UVB irradiation [10]. Loss of keratinocytic RXRα increases melanocytic tumor formation resulting from chemical carcinogenesis [14–16] and enhances the keratinocytic expression of mitogenic factors including Endothelin-1 (EDN1), Hepatocyte Growth Factor (HGF) and Stem Cell Factor (SCF) [13, 14, 17]. A progressive loss of keratinocytic RXRα protein was observed through analysis of human melanomas collected at different stages of disease progression starting from benign nevi to melanoma in situ and invasive and metastatic melanomas [14]. Through our previous studies we determined that when RXRα is knocked out in the epidermis in combination with an activated Cyclin-Dependent Kinase 4 (CDK4) homozygous-mutation (R24C|R24C), the bigenic mice exhibited an increase in melanomagenesis when treated with DMBA-TPA, suggesting abnormal RXRα signaling cooperates with a key oncogenic driver [14]. We have also shown that loss of keratinoctyic RXRα in combination with either oncogenic N-RAS (*NRAS* ^*Q61K*^) or activating *Cdk4* ^*R24C/R24C*^ mutations in two separate bigenic models, enhances melanomagenesis through chronic UVB exposure [18].

In this study, we investigated the role of keratinocytic RXRα in spontaneous and acute neonatal UVB induced melanoma formation as this is a more biologically-relevant model since malignant melanoma is epidemiologically associated with acute sun exposure [2]. The CDK4 pathway comprising of signaling components like p16^INK4a^-cyclin D-CDK4/6-retinoblastoma is known to be altered in 90% of human melanomas [19], while 15–20% of melanoma cases show mutations in the *NRAS* gene [20]. We observed that *Rxrα* loss in the epidermal keratinocytes when combined with *Cdk4* ^*R24C/R24C*^ and *NRAS* ^*Q61K*^ mutations culminated in an enhanced number of spontaneous and acute UVB-induced melanocytic lesions relative to the control mice with functional RXRα (*Rxrα* ^*L2/L2*^). Melanomas from the spontaneous and UVB treated groups of trigenic *Rxrα* ^*ep−/−*^ mice were increasingly proliferative and exhibited an increased rate of malignant conversion and tumor vascularization, while apoptotic melanocytes were reduced. The draining lymph nodes of trigenic *Rxrα* ^*ep−/−*^ mice showed an increased number of melanoma cells invasion compared to the controls with functional RXRα. Additionally, the normal skin adjacent to the tumor from both groups of untreated and UVB-treated trigenic *Rxrα* ^*ep−/−*^ mice showed aberrant expression of several key markers of melanoma. In particular, we observed significant increase in the phosphorylated AKT, (active form of Protein Kinase B), p21 and Cyclin D1 and reduced expression of pro-apoptotic marker BAX in the UVB treated trigenic mice, while in the un-irradiated mice we observed a significant increase in expression of p21 and Cyclin D1. Altogether, our results indicate that ablation of keratinocytic RXRα in combination with driver mutations such as *NRAS* ^*Q61K*^ and *Cdk4* ^*R24C/R24C*^, abets the formation of spontaneous melanomas that are malignant in nature and these effects are further exacerbated by a single neonatal UVB exposure.

## Methods

### Mice

Generation of *Rxrα* ^*ep−/−*^ [15], *Cdk4* ^*R24C/R24C*^ [21], and *Tyr-NRAS* ^*Q61K*^ [22] mice have been described previously. See Additional file 1: Figure S1 for breeding strategies for generation of the *Rxrα* ^*ep−/−*^|*Tyr-NRAS* ^*Q61K*^| *Cdk4* ^*R24C/R24C*^ trigenic mice. All of the genotyping PCR primers used in this study are listed in Additional file 2: Table S2. Mice were accommodated in our approved University Animal Facility. These facilities have 12 h light cycles and food/water were provided ad libitum. Animal Care and Use Protocol (ACUP) granted all the institutional approvals for all experiments.

### UVB treatment of mice

P2 mice which were age- and sex-matched (*n* = 12) were subjected to a single 800 mJ/cm2 of UVB light dose from a UV box of four Philips TL-20 W/12RS UV-B [13], which activates the melanocytes to migrate from hair follicles into the extrafollicular epidermis and dermis [13] and leads to melanoma formation [23]. Upon weaning (21 days postnatal), mice were observed weekly for appearance of melanocytic tumors. By 6 months’ post UVB, melanocytic tumors were analyzed and quantified, and biopsies of tumors and tumor adjacent normal (TAN) skin were recovered for further assays. The experiment was independently performed twice with at least six mice in each group of control and mutant mice.

### Histological analyses

5 μm thick formalin-fixed paraffin (FFPE) sections were used for all histological analysis. Hematoxylin and eosin (H&E) staining was performed as previously described [16].

### Immunohistochemistry

All IHC studies, (fluorescent and chromogenic) were done on FFPE sections that were 5 μm in thickness. Prior to staining, melanin pigment was bleached by treating slides with 5% H2O2 in 1X PBS for 60 min at 55 °C. All the antibodies used are detailed in Additional file 2: Table S1. For fluorescent TUNEL staining, the DeadEnd TUNEL System (Promega, no. TB235) was used as described [13]. A single section on the same slide without primary antibody was used as a negative control and all experiments were performed in triplicates. See Additional file 2: for a more in-depth protocol.

### Imaging and quantitation of histological experiments

Leica DME light microscope equipped with its own software, version 3.3.1 was used to capture brightfield images. Whereas, a Zeiss AXIO Imager.Z1 was used to capture fluorescent images, which were analyzed with help of AxioVision 4.8 and Adobe Photoshop. Images from multiple random fields (~ 12–15) were captured from replicate mice in all groups, positive cell were counted and quantified using the ImageJ software (NIH). The slides were analyzed independently by two investigators in a double-blinded manner and significance was determined using a Student’s two-tailed t-test as calculated by GraphPad Prism software.

### Immunoblotting analyses

Standard protocols were used to perform all immunoblotting analyses (described in [13, 24]). After incubation with appropriate secondary antibody, signals were detected using immuno-chemiluminescent reagents (GE Healthcare, Piscataway, NJ). β-actin antibody (#A300–491, Bethyl) was used as a protein loading control. See Additional file 2: Supplementary Methods for detailed protocol. All the antibodies used for immuno-blotting has been listed in the Additional file 2: Table S1.

## Results

### Ablation of epidermal *Rxrα* expression in combination with mutated *Cdk4* and oncogenic *NRAS* mutations results in increased spontaneous and acute UVB-induced melanomagenesis

Previously we have mentioned that RXRα protein in the keratinocytes is involved in melanocyte homeostasis and melanomagenesis, in mouse models where *Rxrα* is knocked out in the epidermal region (*Rxrα* ^*ep−/−*^) [13, 14, 16]. Furthermore, loss of keratinocytic *Rxrα* expression when combined with activated *Cdk4* ^*R24C/R24C*^ or oncogenic human *NRAS* (*Tyr-NRAS* ^*Q61K*^) after chronic UVB-irradiation results in malignant melanoma formation [18]. Therefore, our hypothesis was that keratinocytic *Rxrα* ablation when combined with homozygous activated *Cdk4* ^*R24C/R24C*^ and heterozygous expression of human *NRAS* (*Tyr-NRAS* ^*Q61K*^) would result in enhanced formation of spontaneous malignant melanoma, which would be further aggravated/ accelerated by a single neonatal UVB-irradiation in the trigenic mouse model.

To that end, we have combined the *Rxrα* ^*ep−/−*^ mice, in which *Rxrα* was specifically deleted in epidermal keratinocytes, with oncogenic Neuroblastoma RAS Viral Oncogene Homolog (*NRAS* ^*Q61K*^) and activating *Cdk4* ^*R24C/R24C*^ to generate a trigenic mouse line by breeding together our *Rxrα* ^*ep−/−*^ *|Cdk4* ^*R24C/R24C*^ and *Rxrα* ^*ep−/−*^ *|Tyr-NRAS* ^*Q61K*^ bigenic mouse lines. This will allow us to understand the contribution of epidermal RXRα in melanoma formation together with aberrant signaling pathways such as MAPK and CDK4 and after a single neonatal UVB exposure. (see Materials and Methods, Additional file 1: Figure S1a). *Rxrα* ^*L2/L2*^|*Cdk4* ^*R24C/R24C*^|*Tyr-NRAS* ^*Q61K*^ mice (floxed *Rxrα* mice which contain LoxP sites that flank exon 4) were used as “controls” for wild-type *Rxrα*. From here onwards, we will refer to the homozygous *Cdk4* ^*R24C/R24C*^ mutation as *Cdk4* ^*R24C*^. Groups of neonatal (P2) mice from control and trigenic mice were left with no UVB treatment and were monitored for spontaneous melanoma formation, while a second cohort of control and trigenic mice were exposed to a single dose of 800 mJ/cm^2^ UVB at P2 and monitored periodically for formation of melanocytic tumors (Additional file 1: Fig. S1b) [18]. Neonatal UVB dose stimulates migration of melanocytes into the epidermis and dermis and promotes melanomagenesis. [13, 23]. Melanocytic tumors in the UVB treated trigenic mice developed within 6–7 months of UVB treatment, while the spontaneous melanocytic lesions appeared approximately a year after birth. Here we have characterized and compared the melanocytic tumors in the *Rxrα* ^*ep−/−*^|*Cdk4* ^*R24C/R24C*^|*Tyr-NRAS* ^*Q61K*^ mice induced by a single neonatal UVB exposure after 6 months of incubation, with the spontaneous melanocytic lesions that develop in the 12 month old *Rxrα* ^*ep−/−*^|*Cdk4* ^*R24C/R24C*^|*Tyr-NRAS* ^*Q61K*^ mice.

**Fig. 1:**
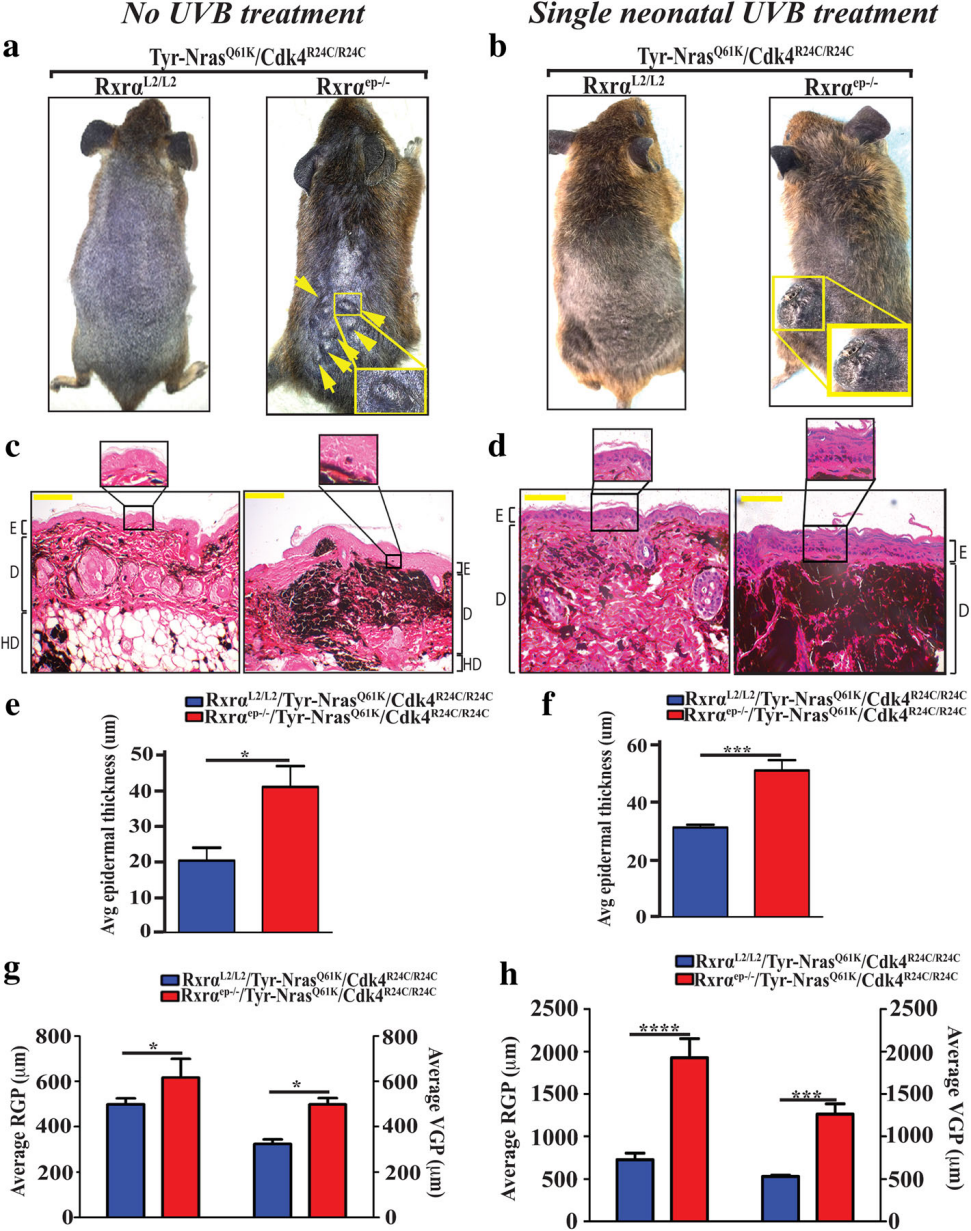
Macroscopic and histological characterization of spontaneous and acute UVB induced melanomas from control and RXRα^ep−/−^|TyrNRAS ^Q61K^|Cdk4^R24C/R24C^ mice. Mice with epidermal-specific Rxrα ablation in combination with Tyr-NRAS^Q61K^ and homozygous Cdk4^R24C^ mutations have (**a**) spontaneous melanocytic growths; (**b**) larger melanocytic tumors with some degree of ulceration post single neonatal UVB treatment compared to mice with functional Rxrα. Lesions indicated by arrows. Histological analyses of melanocytic tumors from Tyr-NRAS^Q61K^ and Cdk4^R24C^ mice and with functional (left panel) and ablated (right panel) Rxrα. Tyr-NRAS^Q61K^ */*Cdk4^R24C^ mice with epidermal-specific Rxrα ablation have (**c**) more pigmented lesions with minimal penetration into epidermal basal layer (inset); (**d**) greater degree of densely pigmented lesions with enhanced penetration into epidermal basal layer after acute neonatal UVB treatment. E, epidermis; D, dermis; HD, hypodermis. Scale bar =50 μm. **e, f** Increased epidermal thickness, and (**g, h**) higher radial growth phase (RGP) and vertical growth phase (VGP) of melanocytic tumors in RXRα^ep−/−^ |Tyr-NRAS^Q61K^ |Cdk4^R24C/R24C^ mice with (**e, g**) no UVB treatment and (**f, h**) single UVB treatment relative to their respective controls. Statistical relevance indicated as follows; * = *p* < 0.05

Phenotypically, overall skin of the control and trigenic mice was heavily pigmented throughout (Fig. 1a), which made it difficult to quantitate smaller melanocytic lesions. *Rxrα* ^*ep−/−*^|*Cdk4* ^*R24C/R24C*^|*Tyr-NRAS* ^*Q61K*^ mice with larger lesions were easier to quantitate, as they were raised at that size. Strikingly, UVB induced *Rxrα* ^*ep−/−*^|*Cdk4* ^*R24C/R24C*^|*Tyr-NRAS* ^*Q61K*^ mice developed significantly larger melanocytic tumors with some degree of ulceration compared to the untreated trigenic mice (Fig. 1a, b). To further characterize the bigenic “control” and trigenic mice, we performed hematoxylin and eosin (H&E) staining on paraffin sections with thickness of 5 μm on the dorsal skin from both genotypes. The melanocytic lesions were more densely pigmented (Fig. 1c, d), and were highly penetrant into the epidermal basal layer (Fig. 1c, d (inset)) and exhibited a significant increase in epidermal thickness in the skin of *Rxrα* ^*ep−/−*^|*Cdk4* ^*R24C/R24C*^|*Tyr-NRAS* ^*Q61K*^ mice compared to *Rxrα* ^*L2/L2*^|*Cdk4* ^*R24C/R24C*^|*Tyr-NRAS* ^*Q61K*^ mice, although UVB seemed to exacerbate the effects (Fig. 1e, f). This phenotype is quite often seen in mice with an epidermal ablation of RXRα, as the keratinocytes present in the basal layer are hyperproliferative [15]. We observed a significantly increase in both the radial growth phase (RGP) and vertical growth phase (VGP) of the melanocytic tumors in the dermis in the trigenic mice compared to the control in the untreated mice (Fig. 1g) while the UVB treated trigenic mice showed significantly heightened increase in the RGP and VGP relative to their control bigenic mice (Fig. 1h). Histopathological analyses confirmed that the melanocytic lesions from the no UVB treated trigenic mouse line are melanomas with hallmark features like round and/or spindle cell tumors and mild to moderate anisokaryosis, which is the variation in size of nuclei in excess of the normal range for a tissue. Meanwhile the lesions from the acute UVB treated trigenic mice are anaplastic melanomas with poor differentiation and moderate to marked anisokaryosis [5 times the normal nuclei size] (Additional file 1: Fig. S1c). Altogether, above results suggest that loss of epidermal *Rxrα* when combined with oncogenic NRAS and activated CDK4 results in melanomagenesis, which is further accelerated by a single neonatal UVB exposure reducing the tumor latency as shown in the Kaplan Meier curve (Additional file 3: Fig. S2a).

### Increased proliferation, malignant conversion, enhanced vascularization and reduced apoptosis in melanomas from trigenic mice lacking keratinocytic *Rxrα* expression in the epidermis

**Fig. 2:**
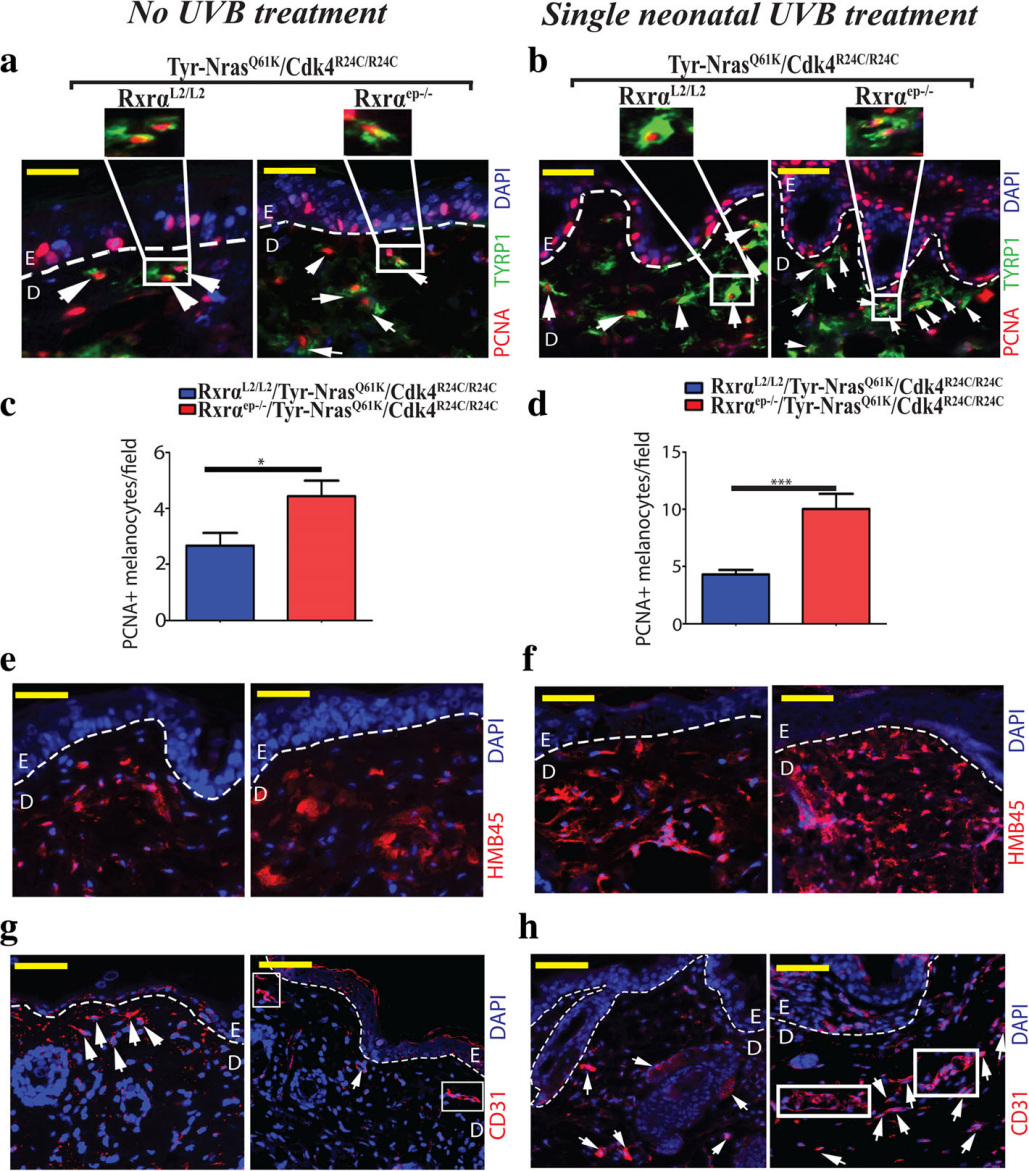
Melanomas from *RXRα* ^*ep−/−*^|*TyrNRAS* ^*Q61K*^|*Cdk4* ^*R24C/R24C*^ mice display enhanced proliferation, malignant conversion and angiogenesis relative to their controls. **a, b** Fluorescent IHC for proliferation marker PCNA (red) and melanocyte marker TYRP1 (green). A trend in overall increase of PCNA+/TYRP1+ cells was observed in lesions from Rxrα^ep−/−^ mice compared to Rxrα^L2/L2^ controls in combination with Tyr-NRAS^Q61K^ and Cdk4^R24C^ mutations in no UVB treated mice (**a**) and single neonatal UVB treated mice (**b**). **c, d** Bar-graph represents PCNA + |TYRP2+ melanocytes/ field in no UVB and acute UVB treatment respectively. **e, f** IHC using antibody cocktail of antibodies directed against malignant melanoma antigens HMB45 and MART-1 (red). Overall, more positive staining was observed in lesions from the triple knockout mice with *RXRα* ^*ep−/−*^ ablation compared to bigenic mice with their respective Rxrα^L2/L2^ controls mutations in no UVB treated mice (**e**) and single neonatal UVB treated mice (**f**). **g, h** IHC for tumor angiogenesis marker CD31 (red). Overall, more prominent staining was observed in lesions from trigenic *RXRα* ^*ep−/−*^ mice compared to controls. Loss of epidermal Rxrα results in lesions with multicellular CD31+ blood vessels (**g**, right panel) in mice with no UVB treatment while acute UVB treated mice resulted in larger multicellular CD31+ blood vessels (**h**, right panel). **a, b, c, d, e, f, g, h** White dashed lines, artificially added, indicate epidermal-dermal junction. Blue color corresponds to DAPI staining of the nuclei. E = Epidermis, D = Dermis. Scale bars = 50 μm. Statistical relevance indicated as follows; * = p < 0.05

**Fig. 3:**
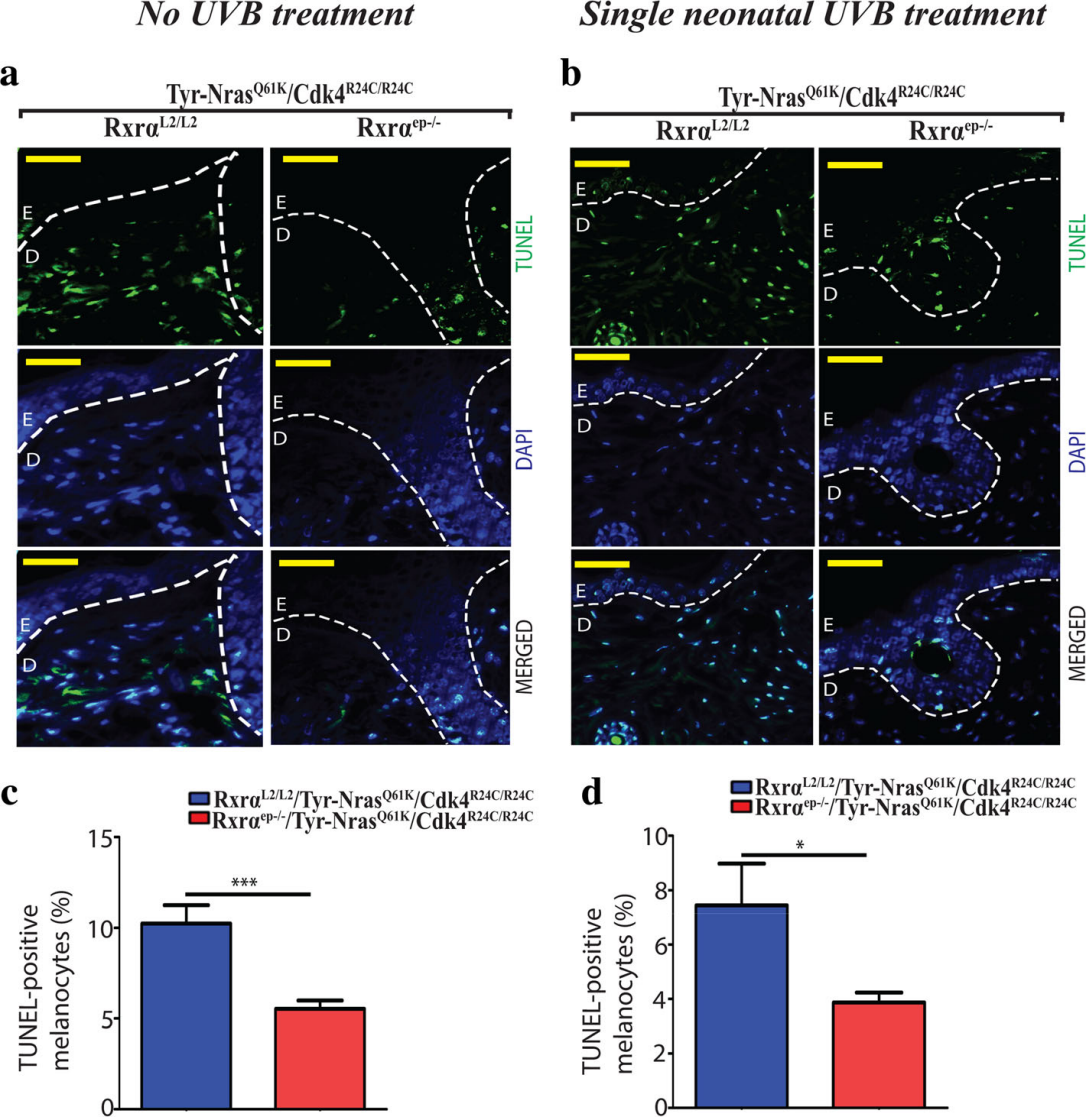
Melanomas from trigenic *RXRα* ^*ep−/−*^|*TyrNRAS* ^*Q61K*^|*Cdk4* ^*R24C/R24C*^ mice display reduced apoptosis relative to their controls with functional RXRα. **a, b** TUNEL assay to label apoptotic cells. Apoptotic cells are indicated by green staining (top panel), blue color corresponds to DAPI staining of the nuclei (middle panel) and merged TUNEL and DAPI cells (lower panel). Overall, reduced TUNEL positive staining was observed in lesions from trigenic Rxrα^ep−/−^ mice compared to their respective controls in mice with (**a**) no UVB treatment as well as the (**b**) acute UVB treated mice. **c, d** Bar-graph represents TUNEL+ melanocytes/ field in no UVB and acute UVB treatment respectively

To further characterize the melanocytic tumors from the trigenic mice, we performed IHC analyses using specific antibodies for markers of proliferation, malignant conversion and vascularization. We aimed to elucidate the differences between melanomas from *Rxrα* ^*ep−/−*^|*Cdk4* ^*R24C/R24C*^|*Tyr-NRAS* ^*Q61K*^ and their corresponding *Rxrα* ^*L2/L2*^ controls from untreated mice at 12 months of age and 6 months after single neonatal UVB treatment as discussed above. In order to determine the proliferation index of the melanocytic tumors, we co-labeled with antibodies for melanocyte-specific enzyme tyrosinase related protein 1 (TYRP1) and PCNA, the marker for proliferation (Fig. 2a, b) [25, 26]. There was a significantly higher number of PCNA+/TYRP1+ co-labeled proliferating melanocytes in the spontaneous melanocytic lesions from trigenic *Rxrα* ^*ep−/−*^ mice as compared to the corresponding control (See Fig. 2c). A further increase in proliferation was observed in the UVB treated trigenic mice compared to their respective controls (Fig. 2d). In order to determine the rate of malignant conversion of the melanocytic tumors, we performed IHC using a cocktail of antibodeies directed against melanoma antigens MART-1 and HMB45 [27]. The malignant melanoma antibody cocktail showed increased staining in the melanocytic tumors from the trigenic *Rxrα* ^*ep−/−*^ mice compared to their control mice in the untreated group (Fig. 2e). Furthermore, UVB treated groups displayed intense staining compared to the no UVB treated mice, which suggested that a larger population of malignant and aggressive cells were present in those melanomas (Fig. 2e, f). We then labeled for the endothelial cell-specific marker Cluster of Differentiation 31 (CD31) (Fig. 2g, h) [28]. Mice from the spontaneous (no UVB) and single UVB treated control groups exhibited a similar number of CD31+ cells compared to the trigenic *Rxrα* ^*ep−/−*^ mice. However, there was a presence of complex CD31+ vasculature, which were larger and present comparatively at a higher incidence in the UVB treated trigenic *Rxrα* ^*ep−/−*^ mice (Fig. 2h, right panel). Altogether, above results indicate that abrogating *Rxrα* expression in the epidermis in combination with activated CDK4 and N-RAS contributes to enhanced proliferation, malignant conversion, and vascularization of melanomas, and exposure to a single neonatal dose of UVB accelerates this process. To detect DNA strand breaks, terminal deoxynucleotidyl transferase-mediated dUTP nick end labeling (TUNEL) assay was used, which is often associated with apoptosis (Fig. 3a, b) [29]. The percentage of dermal TUNEL positive cells in trigenic mice was reduced compared to the controls both in the untreated and single UVB treated group (Fig. 3c, d) suggesting that keratinocytic RXRα plays a role in regulating apoptosis in the dermal melanomas.

### Cutaneous melanomas in the *Rxrα*^*ep−/−*^|*Cdk4*^*R24C/R24C*^|*Tyr-NRAS*^*Q61K*^ trigenic mice metastasize to distal lymph nodes

**Fig. 4:**
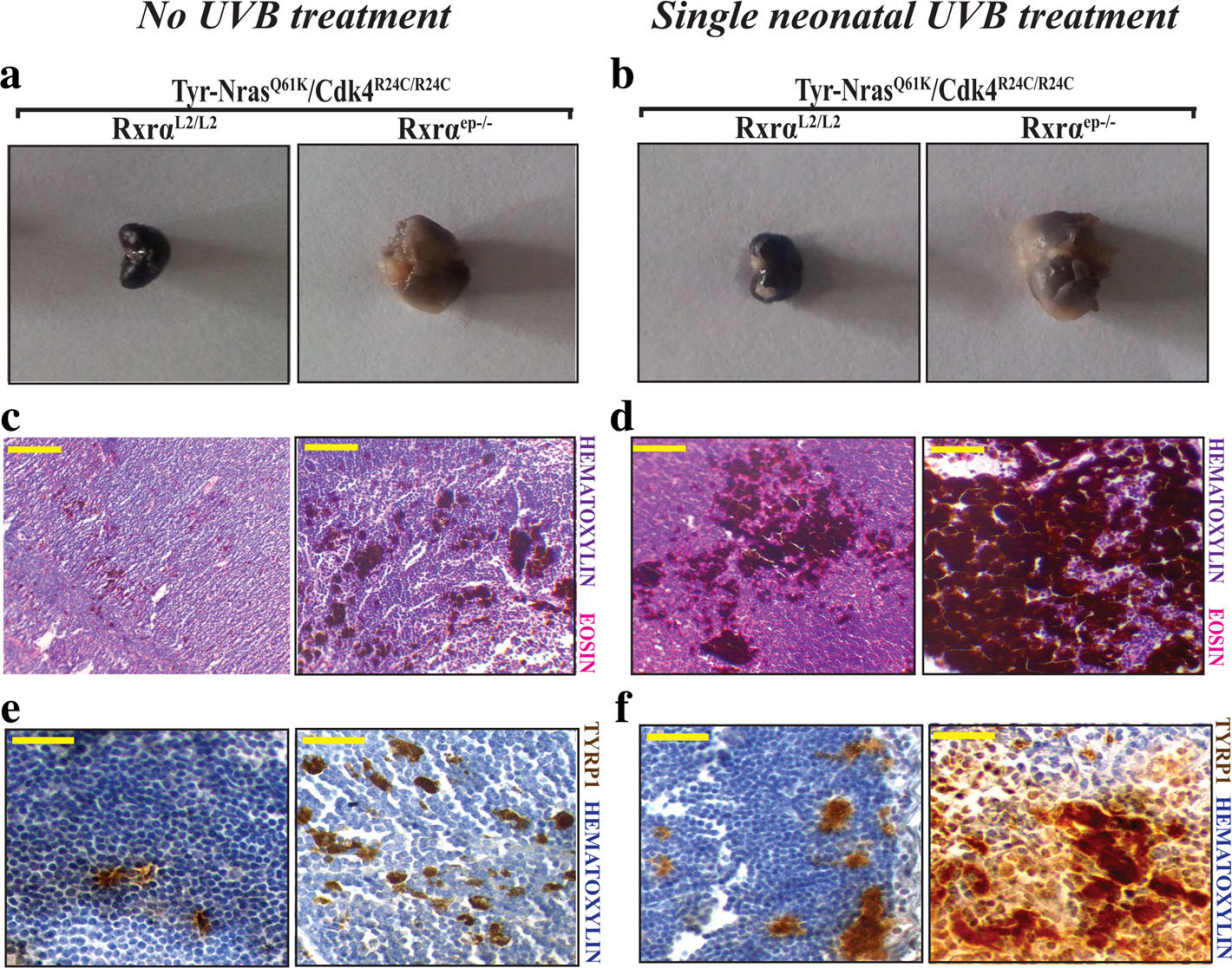
Enhanced metastasis to draining lymph nodes in trigenic *RXRα* ^*ep−/−*^|*Tyr-NRAS* ^*Q61K*^|*Cdk4* ^*R24C/R24C*^ mice relative to *Rxrα* ^*L2/L2*^ control mice. **a, b** Excised draining lymph nodes from RXRα^L2/L2^/Tyr-NRAS^Q61K^/Cdk4^R24C/R24C^ and RXRα^ep−/−^/TyrNRAS ^Q61K^/Cdk4^R24C/R24C^ mice at age (**a**) 12 months for untreated mice and (**b**) 6 months for single UVB treated mice. **(c, d)** Histological analyses of draining lymph nodes from Tyr-NRAS^Q61K^/Cdk4^R24C^ mice with functional and ablated *Rxrα* in (**c**) no UVB treatment as well as the (**d**) acute UVB treated mice. **e, f** Chromogenic IHC for melanocyte-specific marker TYRP1 (brown). More positive staining overall is observed in RXRα^ep−/−^/TyrNRAS ^Q61K^/Cdk4^R24C/R24C^ LNs as opposed to their relative RXRα^L2/L2^ control LNs in both the UVB untreated and treated groups. Hematoxylin (purple) was used as a nuclear counterstain. Scale bar =100 μm

In order to understand what effects does loss of keratinocytic *Rxrα* have on metastasis of spontaneous and UVB induced melanomas, the axillary and inguinal draining lymph nodes (LNs) from *Rxrα* ^*ep−/−*^|*Cdk4* ^*R24C/R24C*^|*Tyr-NRAS* ^*Q61K*^ mice were analyzed for the presence of invasive melanocytes and compared them with lymph nodes from *Rxrα* ^*L2/L2*^|*Cdk4* ^*R24C/R24C*^|*Tyr-NRAS* ^*Q61K*^ (control) mice. Lymph nodes from the trigenic mice were enlarged compared to their respective control mice in both the UVB untreated (spontaneous) and treated groups, as there was more drainage in the mice where *Rxrα* was ablated in the epidermal keratinocytes (Fig. 4a, b). To further characterize the lymph nodes of the control and trigenic mice, we performed hematoxylin and eosin (H&E) staining paraffin sections of the LNs from both the UVB untreated (spontaneous) and single UVB treatment groups (Fig. 4c, d). An increased number of pigment-containing cells in LNs from *Rxrα* ^*ep−/−*^|*Cdk4* ^*R24C/R24C*^|*Tyr-NRAS* ^*Q61K*^ mice was observed compared to the control mice in the group with spontaneous melanomas (Fig. 4c). Meanwhile, the trigenic mice in the single neonatal UVB treated group showed deep and highly pigmented regions compared to their corresponding controls (Fig. 4d). The presence of melanocytic cells in the LNs was analyzed by performing IHC staining for TYRP1 on LN sections from both mutant and control mice (Fig. 4e, f). We observed a significantly higher number of cells positive for TYRP1 in the trigenic group compared to the bigenic group with spontaneous melanomas (Fig. 4e), and a significant increase in TYRP1+ cells in LNs from the *Rxrα* ^*ep−/−*^ mice compared to their *Rxrα* ^*L2/L2*^ controls post acute neonatal UVB exposure (Fig. 4f). These results indicate that knocking out of epidermal *Rxrα* expression in combination with oncogenic NRAS and activated CDK4 results in formation of spontaneous and UVB-induced melanomas with a hightened chance of metastasis of melanoma cells to the distal LNs.

### Altered melanoma signaling in tumor adjacent normal (TAN) skin from spontaneous and acute UVB irradiated mice with loss of *Rxrα* expression in the keratinocytes

Since keratinocytic *Rxrα* ^*ep−/−*^ mutant mice formed spontaneous and acute neonatal UVB-induced melanocytic lesions relative to control mice with functional *RXRα*, our next aim was to understand if removal of keratinocytic *Rxrα* also leads to phenotypic changes in the normal skin adjacent to the tumor that increases susceptibility to melanomas. To help characterize that, we performed immunoblotting analyses to determine the changes in the expression of known key biomarkers of melanoma susceptibility on whole skin biopsies of tumor adjacent normal (TAN) skin from the spontaneous (no UVB) and single neonatal UVB-irradiated *Rxrα* ^*ep−/−*^ mutant mice and compared them to their controls. The TAN skin had no evidence of melanoma cells as observed from the histopathological analyses of both age matched non-UVB and UVB treated skin from bigenic control and trigenic mice (Additional file 3: Fig. S2b, c).

**Fig. 5:**
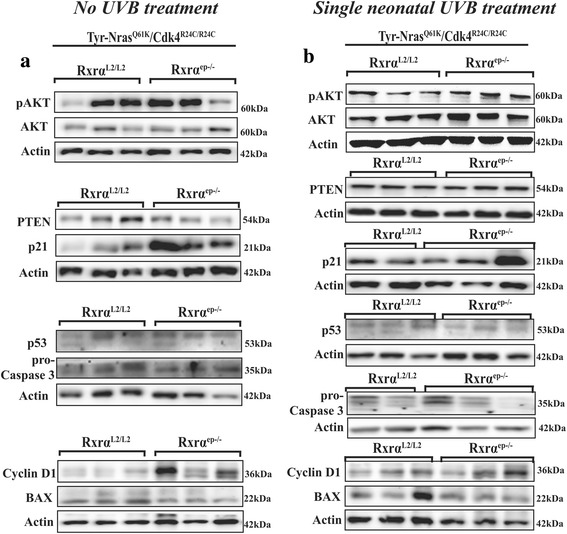
Spontaneous and acute UVB-irradiated skin from trigenic mice exhibit altered expression of regulators of signaling. **a** Immumnoblotting analyses of non-UV irradiated TAN skin samples showed modest upregulation in pAKT, significant upregulation of p21 and Cyclin D1 and a modest decrease in BAX expression in the *Rxrα* ^*ep−/−*^ mutant mice relative to the controls in the untreated group. **b** Western blot analysis of single UVB treated groups of control and trigenic *Rxrα* ^*ep−/−*^ mutant mice exhibiting significant upregulation of pAKT, p21 and Cyclin D1 expression and a significant decrease in BAX expression in the trigenic mice relative to the controls. Equal loading was confirmed by β-actin. 2–3 biological replicates for each group are shown for all immunoblots

Our data indicates that ablation of keratinocytic *Rxrα* expression resulted in no significant increase in phosphorylation of AKT at Ser473 or total AKT levels in the no UVB treated *Rxrα* ^*ep−/−*^ mutants relative to their *Rxrα* ^*L2/L2*^ controls (see Fig. 5a and Additional file 4: Fig. S3a). No appreciable difference in expression of its upstream regulator PTEN was also observed in the *Rxrα* ^*ep−/−*^ mutants (Fig. 5a and Additional file 4: Fig. S3b). In neonatal UVB treated group, loss of epidermal *Rxrα* expression resulted in a significant increase in expression of Ser473 phosphorylation of AKT with no change in the expression of total AKT and tumor suppressor PTEN (see Fig. 5b and Additional file 5: Fig. S4a, b). Immuno-blotting revealed that expression of p21 and cyclin D1 was significantly increased in *Rxrα* ^*ep−/−*^ mutant mice compared to their *Rxrα* ^*L2/L2*^ control both in the UVB untreated and treated groups, while no change in the expression of p53 was observed (Fig. 5a, b and Additional file 4: Fig. S3c-e, Additional file 5: Fig. S4c-e). Pro-apoptotic protein BAX is moderately decreased in the *Rxrα* ^*ep−/−*^ mutant mice compared to its *Rxrα* ^*L2/L2*^ control in the untreated group and a significant decrease in the treated groups (Fig. 5a, b and Additional file 4: Fig. S3f, Additional file 5: Fig. S4f). No significant changes in the expression of pro-Caspase 3, another apoptosis related protein, was observed in the UVB untreated and treated TAN skin of *Rxrα* ^*ep−/−*^ mutant mice relative to the *Rxrα* ^*L2/L2*^ mice (Figure 5a,b and Additional file 4: Fig. S3 g, Additional file 5: Figure S4 g). Together, these results indicate that ablation of keratinocytic *Rxrα* expression consequences in an alteration of distinct important key signaling pathways involved in the melanoma microenvironment that elevates melanoma susceptibility.

## Discussion

We have previously shown that keratinocytic RXRα has a role in acute UV-induced melanocyte proliferation [13], in chemically induced melanomagenesis [16] and in cutaneous melanoma formation when combined with mutant Cdk4 by chemical carcinogenesis [14]. We have also demonstrated that knocking out of epidermal RXRα in conjunction with oncogenic mutant *NRAS* ^*Q61K*^ or activated *Cdk4* ^*R24C/R24C*^ promotes chronic UVB-induced melanoma formation [18]. As acute neonatal UV exposure is a major risk factor of cutaneous melanoma [2, 23, 30], we herein investigated the consequences of loss of epidermal RXRα in melanoma formation and determined its collaborative role with signaling pathways linked to melanoma driver mutations [*NRAS (Q61K)* and activated *Cdk4 (R24C)*] for the formation of spontaneous and acute UVB-induced melanomas. Our results suggests that keratinocytic RXRα has a protective role against aggressive melanoma formation induced spontaneously or after acute UVB irradiation. In comparison to the bigenic mice containing *RXRα* mutation and oncogenic mutant *NRAS* ^*Q61K*^ or activated *Cdk4* ^*R24C/R24C*^, which didn’t show melanoma formation unless chronically irradiated with UVB for 30 weeks [18], the present trigenic mice (*RXRα* ^*ep/−*^| *Cdk4* ^*R24C/R24C*^| *NRAS* ^*Q61K*^) develop spontaneous melanoma and in the presence of a single neonatal UVB treatment there is a significant reduction in tumor latency and rapid melanoma progression.

*Rxrα* ^*ep−/−*^ mice, in combination with *Tyr-NRAS* ^*Q61K*^ and homozygous *Cdk4* ^*R24C/R24C*^ mutations, developed increased number of spontaneous and acute UVB-induced melanocytic tumors that were also bigger in size with highly penetrating pigmented cells into the basal layer of the epidermis, compared to their corresponding *Rxrα* ^*L2/L2*^ controls. Similar observations were previously made in lesions from our bigenic *RXRα* ^*ep−/−*^|*Tyr-NRAS* ^*Q61K*^ and *RXRα* ^*ep−/−*^ *|Cdk4* ^*R24C/R24C*^ mice when exposed to chronic UVB [18]. In the neonatal mice, when RXRα is ablated in the epidermis, there is an increase in skin hyperplasia, which is reflected by increased epidermal thickness due to proliferation and differentiation as observed in our mutant mice. A significant increase in RGP and VGP is observed when RXRα is lost in the epidermal keratinocytes in the trigenic mice where melanoma formation is seen spontaneously, and a single neonatal UVB exposure aggravates this effect. These observations have been reflected previously in our studies when RXRα^ep−/−^ mice were exposed to acute UVB [13] and when combined with mutant Cdk4 by chemical carcinogenesis [14]. Vertical growth phase is achieved when the nevus acquires both oncogenic and/or mitogenic characteristics, properties that enable cellular proliferation within a foreign matrix, an important step in tumor progression [31]. The increase in PCNA+/TYRP1+ cells within the *RXRα* ^*ep−/−*^ *|Tyr-NRAS* ^*Q61K*^ *|Cdk4* ^*R24C/R24C*^ skin as compared to the *Rxrα* ^*L2/L2*^ control mice indicated the presence of a large volume of proliferating melanocytes in the mutant melanomas in absence or in presence of acute UVB. *RXRα* ^*ep−/−*^ *|Tyr-NRAS* ^*Q61K*^ *| Cdk4* ^*R24C/R24C*^ melanocytic tumors displayed an increase in the number of cells that stained positive for melanoma antibody cocktail HMB45 and MART-1, which correlated well with the higher VGP in those mice, and a similar increase in CD31 staining implies that the larger melanocytic tumors formed in those mice would necessitate additional vascularization for nutritional support. Enhanced expression of melanoma antibody HMB45 cocktail has been correlated with melanocytic tumors and melanomas [27, 32, 33], while CD31 immuno-reactivity is closely linked to melanocytic tumor progression and the presence of its aggressive behavior [34]. In our previous study, both the two bigenic mouse lines *Rxrα* ^*ep−/−*^ | *Tyr-NRAS* ^*Q61K*^ and *Rxrα* ^*ep*−/−^ |*Cdk4* ^*R24C|R24C*^ [18] have increased expression of *angiopoietin 1 (Angpt1)*, which contributes to formation of blood vessels. It is likely that increased angiogenesis in the UV irradiated trigenic mice could be, at least in part, due to increased *Angpt1 expression.* It remains to be determined whether RXRα directly regulate *Angpt1* expression, or whether hypoxia/ altered HIF signaling in the melanoma microenvironment modulates secretion of angiogenic factors and contributes towards enhanced angiogenesis.

Failure to trigger apoptosis is a hallmark of cancer when low apoptotic indices in melanoma are observed, particularly in advanced stages [35]. Interestingly, a reduced percentage of TUNEL+ dermal melanocytes were detected in the mutant melanomas developed spontaneously or after acute UVB irradiation. Melanocytic lesions from DMBA-TPA treated *Rxrα* ^*ep−/−*^ |*Cdk4R24C* mice [14] and in our chronic UVB exposed bigenic mice studies [18] were similarly proliferative, angiogenic and had malignant and metastatic characteristics, although reduction in apoptotic melanocytes in our trigenic *Rxrα* ^*ep−/−*^ mice is unique to the current study. Altogether, above data reiterates that formation of melanocytic tumors in the absence of keratinocytic RXRα, and in the presence of oncogenic NRAS mutation and activated CDK4, have increased metastatic capabilities compared to the control mice with an accelerated effect in the acute UVB treated mice.

There was a higher degree of malignancy in melanomas from the trigenic *Rxrα* ^*ep−/−*^ mouse line, where we observed a higher number of TYRP1-expressing melanoma cells invading into draining lymph nodes in *RXRα* ^*ep−/−*^ *|Tyr-NRAS* ^*Q61K*^|*Cdk4* ^*R24C/R24C*^ mice with increased drainage seen in the UVB treated mice. In our DMBA-TPA treated *Rxrα* ^*ep−/−*^ *|Cdk4* ^*R24C/R24C*^ mice [14] and chronic UVB treated *RXRα* ^*ep−/−*^|*Tyr-NRAS* ^*Q61K*^ and *RXRα* ^*ep−/−*^ *|Cdk4* ^*R24C/R24C*^ bigenic mice [18], we observed similar increase in proliferation, angiogenesis, and malignant/metastatic properties. However, no metastasis was observed in other internal organs like liver, lungs, brain and spleen. These observed results highlight a crucial role for RXRα in mediating melanocyte homeostasis, proliferation, angiogenesis and apoptosis and indicate that ablation of keratinocytic RXRα contributes to the progression of spontaneous and acute neonatal UVB-induced melanocytic tumors to malignant and metastatic tumors. These observations correlate well with our previous studies in human melanomas, which demonstrated that RXRα protein is progressively lost in the epidermal keratinocytes as tumors progress from benign nevi/lesions to aggressive melanomas [14].

We observed an increased expression of p21, a cyclin dependent kinase inhibitor and an upregulation of Cyclin D1 in the TAN skin from trigenic *Rxrα* ^*ep−/−*^, without appreciable difference in p53 expression. Although, we have previously observed a reduced expression of p53 in our chronic UVB exposed *RXRα* ^*ep−/−*^|*Tyr-NRAS* ^*Q61K*^ and *RXRα* ^*ep−/−*^ *|Cdk4* ^*R24C/R24C*^ bigenic mice skin [18] and P21 expression is known to be tightly regulated by p53 [36], p21 can be expressed without being induced by p53 via a p53 independent pathway [37, 38]. At this point, it is uncertain if the lack of p53 downregulation is due to difference in the UV treatment regime (chronic vs acute) between the two studies. It also remains to be determined the presence of p53 somatic mutations in the spontaneous and acute UV induced melanomas, which may lead to loss of function of p53 protein. Progression of primary melanoma has been correlated with increased expression of Cyclin D1 [39, 40] and is upregulated due to inactivation of p53, although like p21, Cyclin D1 can be regulated independent of p53 expression [41].

PTEN functions as a tumor suppressor and the loss of functional PTEN results in AKT activation, suppression of apoptosis, and promotion of tumorigenesis [42]. PTEN and AKT normally share a homeostatic balance, and when PTEN is functionally suppressed results in increased phosphorylation of AKT [42] as observed previously in our chronic UVB exposed bigenic mice [18]. Although, we did not observe any loss of PTEN or an increase in activated, pAKT protein in *Rxrα* ^*ep−/−*^ TAN skin in the spontaneous melanocytic tumors, a significant increase in phosphorylated AKT at Ser 473 was noted in the acute UVB treated *Rxrα* ^*ep−/−*^ TAN skin. Different mechanisms of action may explain those findings in un-irradiated skin, such as the inhibition of phosphorylation of AKT by PTEN may be impaired through its somatic mutation and inactivation, without alteration in protein expression as previously found in a number of malignancies including melanoma [43]. Alternatively, PI3K-independent activation of AKT such as ILK-1 associated phosphorylation of AKT at Ser 473 has been observed previously [44]. AKT activation independent of PI3K is also reported through glucagon-like peptide-1 (GLP-1) and glucose-dependent insulinotropic polypeptide (GIP) [45]. PI3K independent activation of AKT through a diverse group of tyrosine (Ack1/TNK2, Src, PTK6) and serine/threonine (TBK1, IKBKE, DNAPKcs) kinases has been reported previously [46]. PTEN expression was unchanged in breast and ovarian cancers where the constitutively active AKT positive specimens show no alteration in PI3K or PTEN [47].

In addition, we observed a decrease in the expression of pro-apoptotic protein BAX in the UVB untreated *Rxrα* ^*ep−/−*^ TAN skin compared to the controls, which was also reduced in the mutant skin several weeks after exposure to a neonatal single UVB treatment. Similarly, expression of Pro-Caspase-3 was moderately reduced in the acute UVB treated *Rxrα* ^*ep−/−*^ TAN skin, although we were unable to detect expression of cleaved Caspase-3. p53 mediated apoptosis usually involves upregulation of pro-apoptotic Bcl-2 members such as BAX and activation of Caspase-3 [48, 49], and BAX and Caspase 3 can also be activated independent of p53 activation [50, 51]. Reduced expression of BAX and Pro-Caspase 3 in the mutant skin without significant changes in p53 expression could be a p53 independent event as reported earlier. Since the above results are generated from tumor adjacent skin, they firmly ascertain that loss of epidermal RXRα alone or together with acute neonatal UVB create a microenvironment in the skin that is prone to melanomagenesis mediated by driver mutations such as oncogenic NRAS and activated CDK4 mutations. Increased expression of p21 and Cyclin D1 that is observed in the TAN skin of the *RXRα* ^*ep−/−*^ *|Tyr-NRAS* ^*Q61K*^ *|Cdk4* ^*R24C/R24C*^ mice before and after acute UVB treatment, is likely due to the loss of RXRα in the epidermis, thereby contributing to changes in the expression of signaling molecules in the melanoma microenvironment. Acute UVB treatment induces additional alteration including a significant increase in pAKT and a significant decrease in Bax in the tumor microenvironment. It remains to be determined if there is any relationship between the NR signaling pathway and the *BRAF* ^*V600E*^ mutation, which is mutated in >50% of human melanomas [52].

## Conclusions

In summary, we demonstrate an important role of keratinocytic RXRα to (1) suppress the formation of spontaneous and acute UVB-induced melanomas, and (2) prevent their progression to malignancy in combination with activated *CDK4* ^*R24C/R24C*^ and oncogenic *NRAS* ^*Q61K*^. Altogether, RXRα can potentially serve as a clinical prognostic marker and a target for mitigating UV-induced melanoma progression and metastasis in humans, although further studies are necessary in this direction for human relevance using in vitro co-culture techniques with human melanocytes and keratinocytes and in vivo using human xenograft models. Additional studies are also necessary to elucidate the molecular mechanisms underlying the non-cell autonomous role of keratinocyitc RXRα to drive melanomagenesis in the tumor microenvironment.

## Additional files

Additional file 1: Figure S1. Breeding of mouse lines and UV scheme used in this study. (a) Breeding crossings used. K14-Cre^tg/O^ I Rxrα^L2/L2^ is also known as Rxrα^ep−/−^. (b) Scheme for single neonatal UVB treatment of mice. (c) H&E stained section showing poorly differentiated melanoma with abnormally large nuclei compared to the normal range (inset). (TIFF 1999 kb)

Additional file 2: **Table S1.** Antibodies used for immuno-histochemical staining and immuno-blotting with details including the host, detection for-, the source, application and the dilution used. **Table S2.** PCR primers used for genotyping of mouse lines with the forward and reverse primers for K14-Cre, RXRα and CDK4, their sequences and the expected band sizes. Additional Methods. (DOCX 19 kb)

Additional file 3: Figure S2. Tumor latency of the mouse models and histological analyses of TAN skin from un-treated and acute-UVB treated mice. (a) Kaplan-meier curve showing tumor latency in the untreated and UVB treated mice. Reduced tumor latency seen in UVB treated mutant mice compared to UVB untreated mutants. Ticks indicate when a mouse in that group was censored (removed from the study). (b, c) H&E staining of TAN skin of non-UVB treated skin and TAN skin from acute-UVB treated mice. In all groups, TAN skin has similar morphology in both the non-UVB treated skin and single neonatal UVB treated skin, except for increased epidermal thickening; and deeper dermal pigmentation seen in the RXRα^ep−/−^ mice. E = Epidermis, D = Dermis, scale bar =100 μm. (TIFF 2257 kb)

Additional file 4: Figure S3. Changes in the expression of different cellular proteins from the TAN skin in the UVB untreated mice. Quantification of western blot. (a, b) Graphs showing expression of pAKT normalized with AKT and PTEN normalized with actin, no significant change. (c, d, e) Graphs showing expression of p21, cyclin D1 and p53 normalized against actin, with significant increase in p21 (*p* < 0.05) and cyclin D1 (*p* < 0.01) and no significant change in p53. (f, g) Graphs showing expression of bax and pro-caspase 3 normalized against actin, with no significant changes in both bax and pro-caspase 3. (TIFF 273 kb)

Additional file 5: Figure S4. Changes in the expression of different cellular proteins from the TAN skin in the UVB treated mice. Quantification of western blot. (a, b) Graphs showing expression of significant increase in pAKT normalized with AKT (p < 0.05) and PTEN normalized with actin with no significant change. (c, d, e) Graphs showing expression of p21, cyclin D1 and p53 normalized against actin, with significant increase in p21 (p < 0.01) and cyclin D1 (p < 0.05) and no significant change in p53. (f, g) Graphs showing expression of bax and pro-caspase 3 normalized against actin, with significant decrease in bax (p < 0.05) and no significant changes in pro-caspase 3. (TIFF 275 kb)

## Abbreviations

LN: Lymph node
NR: Nuclear receptor
TAN: Tumor adjacent normal
Tyr: Tyrosinase
TYRP1: Tyrosinase-related protein 1
UVB: Ultraviolet B
UVR: Ultraviolet radiation
