# Supplementary figures and images for: Ablation of epidermal RXRα in cooperation with activated CDK4 and oncogenic NRAS generates spontaneous and acute neonatal UVB induced malignant metastatic melanomas
Source: BMC Cancer. 2017 Nov 9;17:736. doi: 10.1186/s12885-017-3714-6 (PMC5679438; doi:10.1186/s12885-017-3714-6)

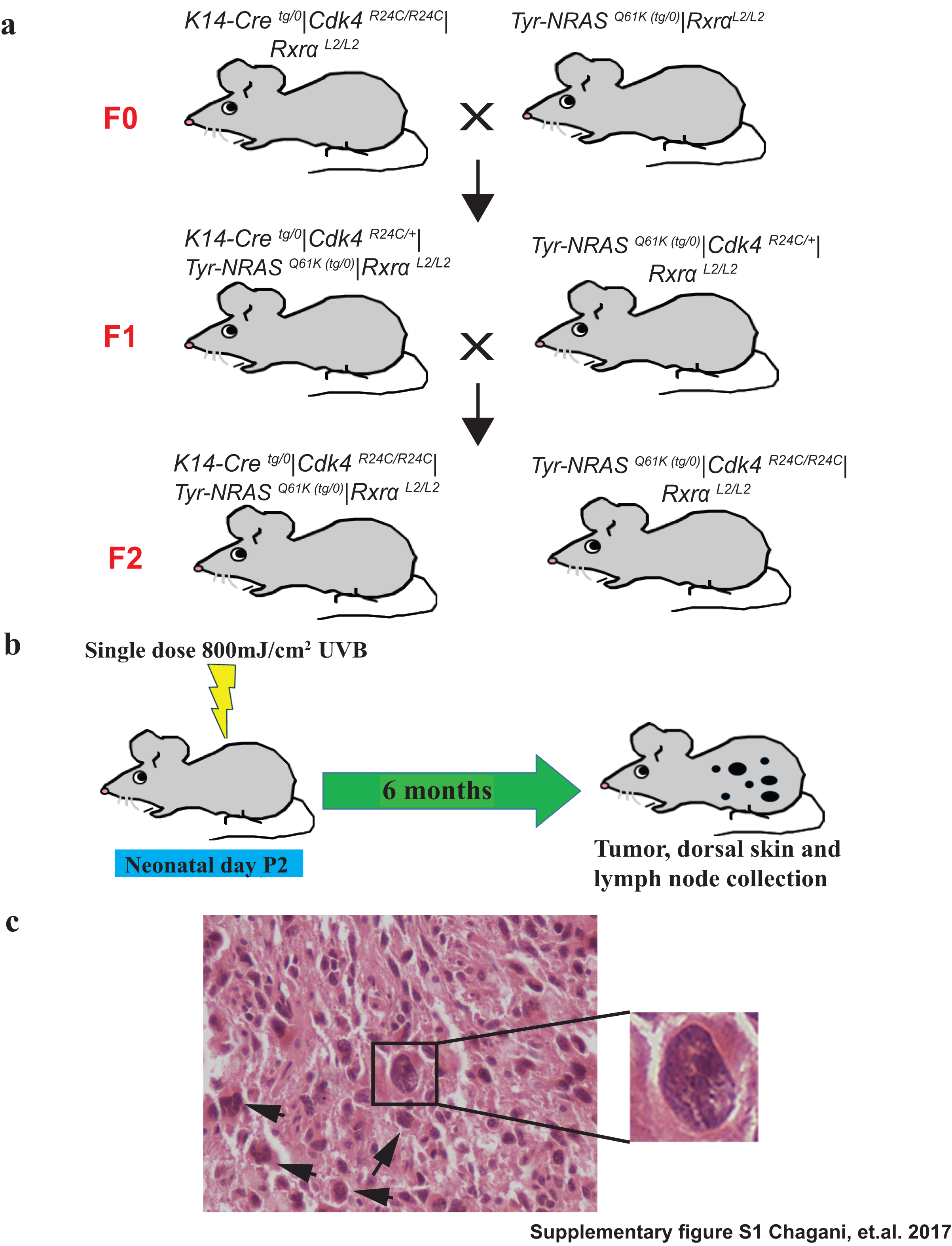

Supplement: Supplementary file 1 — Breeding of mouse lines and UV scheme used in this study. (a) Breeding crossings used. K14-Cretg/O I RxrαL2/L2 is also known as Rxrαep−/−. (b) Scheme for single neonatal UVB treatment of mice. (c) H&E stained section showing poorly differentiated melanoma with abnormally large nuclei compared to the normal range (inset). (TIFF 1999 kb) [file 12885_2017_3714_MOESM1_ESM.tif]

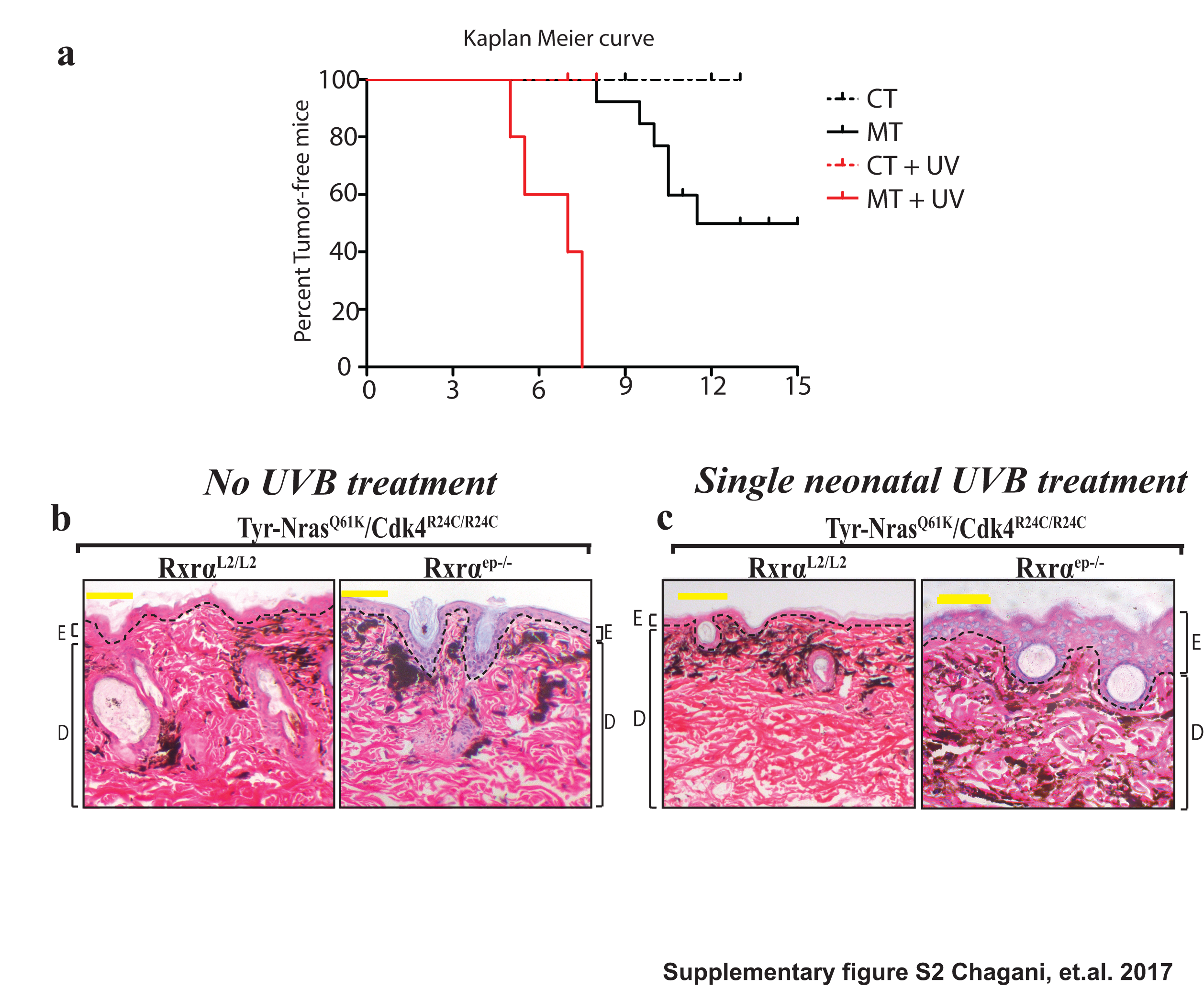

Supplement: Supplementary file 3 — Tumor latency of the mouse models and histological analyses of TAN skin from un-treated and acute-UVB treated mice. (a) Kaplan-meier curve showing tumor latency in the untreated and UVB treated mice. Reduced tumor latency seen in UVB treated mutant mice compared to UVB untreated mutants. Ticks indicate when a mouse in that group was censored (removed from the study). (b, c) H&E staining of TAN skin of non-UVB treated skin and TAN skin from acute-UVB treated mice. In all groups, TAN skin has similar morphology in both the non-UVB treated skin and single neonatal UVB treated skin, except for increased epidermal thickening; and deeper dermal pigmentation seen in the RXRαep−/− mice. E = Epidermis, D = Dermis, scale bar =100 μm. (TIFF 2257 kb) [file 12885_2017_3714_MOESM3_ESM.tif]

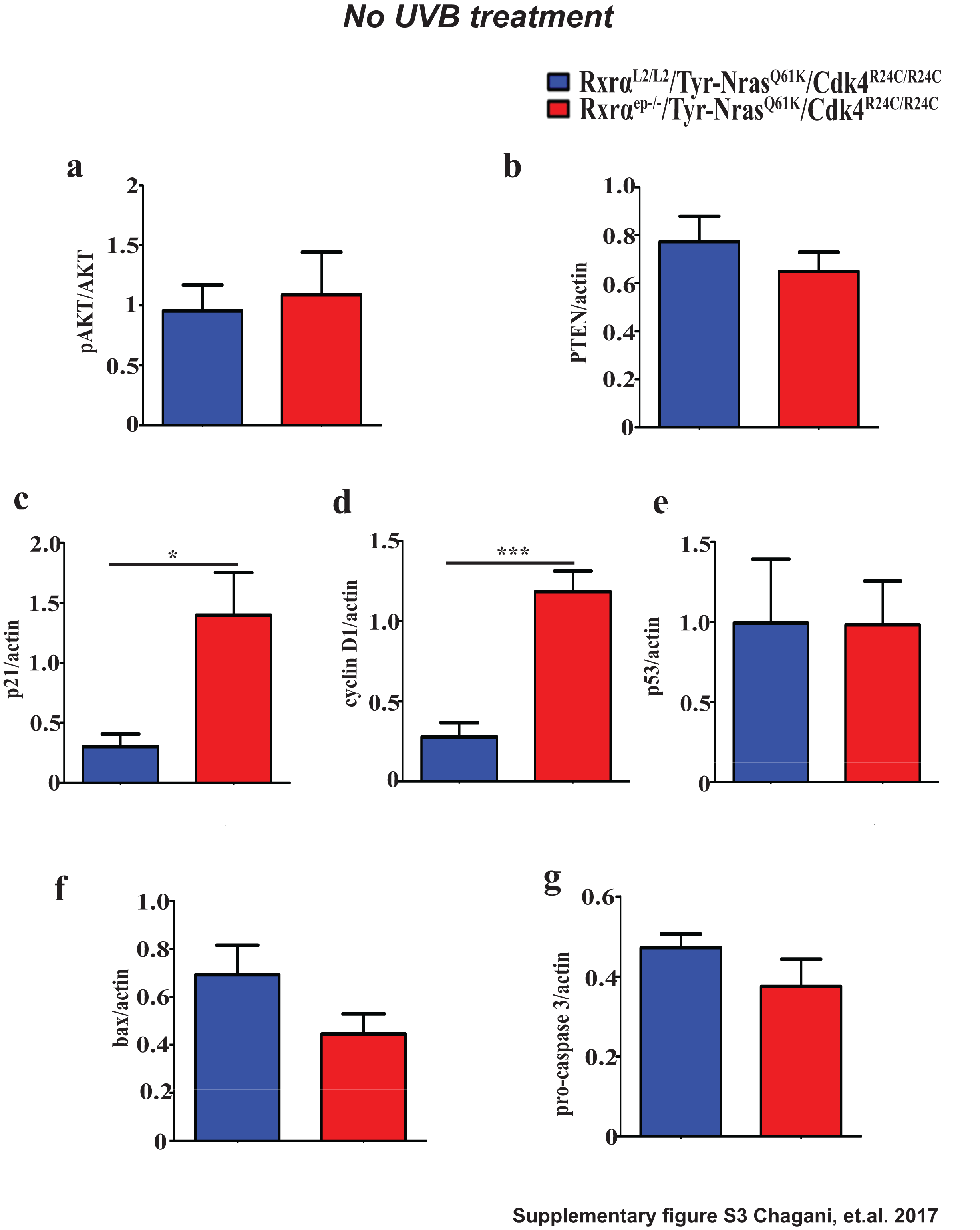

Supplement: Supplementary file 4 — Changes in the expression of different cellular proteins from the TAN skin in the UVB untreated mice. Quantification of western blot. (a, b) Graphs showing expression of pAKT normalized with AKT and PTEN normalized with actin, no significant change. (c, d, e) Graphs showing expression of p21, cyclin D1 and p53 normalized against actin, with significant increase in p21 (p < 0.05) and cyclin D1 (p < 0.01) and no significant change in p53. (f, g) Graphs showing expression of bax and pro-caspase 3 normalized against actin, with no significant changes in both bax and pro-caspase 3. (TIFF 273 kb) [file 12885_2017_3714_MOESM4_ESM.tif]

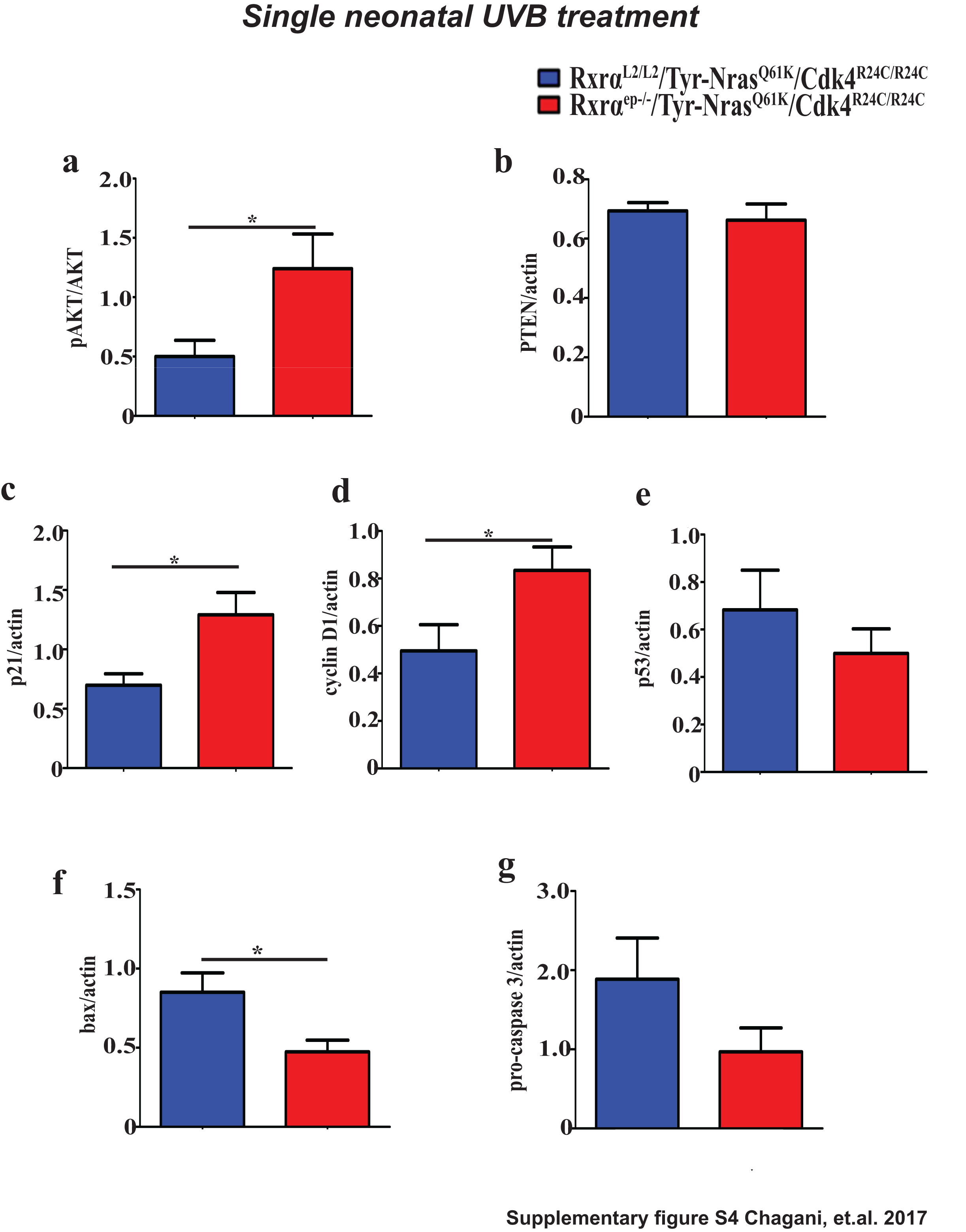

Supplement: Supplementary file 5 — Changes in the expression of different cellular proteins from the TAN skin in the UVB treated mice. Quantification of western blot. (a, b) Graphs showing expression of significant increase in pAKT normalized with AKT (p < 0.05) and PTEN normalized with actin with no significant change. (c, d, e) Graphs showing expression of p21, cyclin D1 and p53 normalized against actin, with significant increase in p21 (p < 0.01) and cyclin D1 (p < 0.05) and no significant change in p53. (f, g) Graphs showing expression of bax and pro-caspase 3 normalized against actin, with significant decrease in bax (p < 0.05) and no significant changes in pro-caspase 3. (TIFF 275 kb) [file 12885_2017_3714_MOESM5_ESM.tif]
