# Supplementary material for: Ablation of epidermal RXRα in cooperation with activated CDK4 and oncogenic NRAS generates spontaneous and acute neonatal UVB induced malignant metastatic melanomas
Source: BMC Cancer. 2017 Nov 9;17:736. doi: 10.1186/s12885-017-3714-6 (PMC5679438; doi:10.1186/s12885-017-3714-6)
Supplement: Supplementary file 2 — Table S1. Antibodies used for immuno-histochemical staining and immuno-blotting with details including the host, detection for-, the source, application and the dilution used. Table S2. PCR primers used for genotyping of mouse lines with the forward and reverse primers for K14-Cre, RXRα and CDK4, their sequences and the expected band sizes. Additional Methods. (DOCX 19 kb) [file 12885_2017_3714_MOESM2_ESM.docx]

**Table S1.** Antibodies used for immuno-histochemical staining and immuno-blotting with details including the host, detection for-, the source, application and the dilution used.

| **Antibody** | **Host** | **For Detection of:** | **Source** | **Application** | **Dilution** |
| --- | --- | --- | --- | --- | --- |
| anti-HMB45 | Mouse | Malignant Melanoma | Abcam (ab732) | IHC-P | 1:100 |
| anti-pAKT | Rabbit | Phosphorylated AKT Protein | cell signaling (4060) | WB | 1:2000 |
| anti-PTEN | Rabbit | PTEN Protein | cell signaling (9559) | WB | 1:1000 |
| anti-p53 | Rabbit | p53 Protein | Santa Cruz (sc-6243), Leica (NCL-p53-CM5p) | WB | 1:500 |
| anti-AKT | Rabbit | Total AKT Protein | cell signaling (6727) | WB | 1:1000 |
| anti-cyclinD1 | Mouse | Cyclin D1 Protein | cell signaling (2926) | WB | 1:2000 |
| anti-B-actin | Rabbit | Loading Control | Bethyl lab (A300-491A) | WB | 1:5000 |
| anti-CD31 | Rabbit | Tumor Angiogenesis | Abcam (ab28364) | IHC-P | 1:50 |
| anti-PEP1 (TYRP1) | Rabbit | Melanocytic cells | NIH (kindly provided by V. Hearing) | IHC-P | 1:1000 |
| anti-PCNA | Mouse | Proliferating Cells | Abcam (ab29) | IHC-P | 1:6000 |
|  |  |  |  |  |  |
| ***IHC-P = Immunohistochemistry - Paraffin Sections, WB = Western Blot*** | | | | | |

**Table S2**. PCR primers used for genotyping of mouse lines with the forward and reverse primers for K14-Cre, RXRα and CDK4, their sequences and the expected band sizes.

| **Gene** | **Primer Name** | **Sequence (5'-3')** | **Expected Bands** |
| --- | --- | --- | --- |
| K14- Cre | TK139 (Forward) | ATTTGCCTGCATTACCGGTC | 349 bp (Cre+), No amplification (Cre-) |
|  | TK141 (Reverse) | ATCAACGTTTTGTTTTCGG |  |
| Rxrα | UN128 (Forward) | CTCAAGTGAGGTGGACATTA | 203 bp (L2), 110 bp (L-) |
|  | WH231 (Reverse) | GAGCTATTGTGCCCTGGAAG |  |
|  | WH233 (L-) | GGAAGCCCAAGATGACCCTC |  |
| Cdk4 | BAB493 (Forward) | TGAAGTGCAGAATCTTCGGTGCAAA | 440 bp (WT), 530 bp (R24C) |
|  | BAB496 (Reverse) | CATTAGGAACTCTCACACTCTTGAG |  |

**Supplementary Methods**

**Histological analyses (Detailed)**

Analyses were performed on 5 µm formalin-fixed paraffin sections. Prior to all procedures, sections were deparaffinized in xylene and rehydrated using graded alcohols. Sections were stained with hematoxylin and eosin (H&E) as previously described [13]. Slides were cleared in xylene and mounted in DPX mounting medium.

**Immunohistochemistry (Detailed)**

5 µm thick sections were deparaffinized and rehydrated as described above. All slides were treated with 10% H_2_O_2_ in 1X PBS as described above to remove melanin pigment. Antigen retrieval was performed in a hot water bath using either Tris-EDTA buffer (pH 9.0) for use with metastatic melanoma antibody cocktail (HMB45 + MART-1) or citrate buffer (pH 6.0) for use with all other antibodies. All sections were washed 3X with 0.05% PBS-Tween (PBST) and treated with 10% normal goat serum in PBST to block nonspecific antibody binding. Sections were then incubated overnight with primary antibody at 4°C. Primary antibody incubation was followed by 3X PBST washes before addition of either biotin or fluorophore-conjugated secondary antibodies. For fluorescent IHC, nuclei were counterstained with 200 ng/mL DAPI (in PBST) for 10 mins. For chromogenic IHC, sections were incubated with streptavidin-horseradish peroxidase (Vector Laboratories), signal developed with DAB peroxidase substrate kit (Vector Laboratories), and counterstained with hematoxylin (1:1 in H_2_0). Finally, sections were rinsed 3X with PBST (fluorescent IHC) or running tap water (chromogenic IHC), dehydrated through sequential alcohol washes and then cleared in xylene. Slides were mounted with DPX mounting medium. Antibodies used are detailed in Supplementary Table 1. Sections on the same slide labeled without primary antibody was used as negative controls, and all experiments were performed in triplicates.

**Immunoblotting analyses (Detailed)**

Protein lysates were obtained by homogenizing tissue in lysis buffer (described in [10, 21]) followed by sonication. Protein concentration was performed using the BCA assay (Thermo Scientific). Protein lysates were resolved using SDS-PAGE gel electrophoresis and transferred onto a nitrocellulose membrane. The blots were blocked overnight with 5% non-fat dry milk and incubated with specific antibodies (detailed in Supplementary Table S1). After incubation with appropriate secondary antibody, signals were detected using immunochemiluminescent reagents (GE Healthcare, Piscataway, NJ). Equal protein loading in each lane was confirmed with a β-actin antibody (#A300-491, Bethyl).
